# Modified minimally invasive transforaminal lumbar interbody fusion using a trans-multifidus approach: a safe and effective alternative to open-TLIF

**DOI:** 10.1186/s13018-015-0234-4

**Published:** 2015-06-12

**Authors:** Wenzhi Zhang, Xu Li, Xifu Shang, Xiang Xu, Yefeng Hu, Rui He, Liqun Duan, Xiaodong Ling, Feng Zhang

**Affiliations:** Department of Orthopaedics, Anhui Medical University affiliated Provincial Hospital, Hefei, 230001 China

**Keywords:** Trans-multifidus approach, Lumbar fusion, Minimally invasive techniques, Modified minimally invasive transforaminal lumbar interbody fusion

## Abstract

**Background:**

Application of minimally invasive transforaminal lumbar interbody fusion (MI-TLIF) is limited by long fluoroscopy time and a steep learning curve. Herein, MI-TLIF was modified using a trans-multifidus approach, assisted by microscope, termed MMI-TLIF, and the clinical outcomes of MMI-TLIF and open-TLIF were compared.

**Methods:**

Forty-nine patients treated with MMI-TLIF were matched with 49 subjects who underwent open-TLIF. Patients were assessed using the North American Spine Society Score (NASS), Oswestry Disability Index (ODI), Short Form-36 (SF-36), and Visual Analogue Score (VAS) before surgery and during follow-up (6 months and 2 years). The four-type Bridwell anterior fusion grading system was used to evaluate fusion rates at 2 years.

**Results:**

The median fluoroscopic time did not differ significantly between the MMI-TLIF and open-TLIF groups. MMI-TLIF surgery took significantly longer than open-TLIF (91.3 vs. 82.5 min; *P* < 0.05). Meanwhile, MMI-TLIF patients lost significantly less blood than open-TLIF patients (75.3 vs. 215.2 ml; *P* < 0.05), and MMI-TLIF patients were hospitalized for less long than open-TLIF patients (3.7 vs. 6.9 days; *P* < 0.05) and reported less pain, faster ambulation, and lower morphine intake than open-TLIF patients (all *P* < 0.05). The NASS, ODI, VAS, and SF-36 scores were significantly improved 6 months and 2 years postsurgery in both groups, compared with preoperative values, and similar values were obtained for both groups. Finally, fusion rates were similar in MMI-TLIF and open-TLIF patients.

**Conclusions:**

Overall, these findings strongly suggest the superiority of MMI-TLIF to open-TLIF. Therefore, MMI-TLIF could be a safe and effective alternative to MI-TLIF and open-TLIF.

## Background

Degenerative disc disease is one of the most common causes of low back pain worldwide. Among a variety of approaches [1, 2], open transforaminal lumbar interbody fusion (open-TLIF) is one of the treatments of choice for a variety of degenerative lumbar disorders. Open-TLIF achieves good fusion rates, maintains the intervertebral space and foraminal dimension, and restores vertebral alignment [3–5]. However, this technique requires an extensive amount of soft tissues to be dissected for pedicle screw insertion and facet complex resection. Unfortunately, the significant damage this causes to muscles and soft tissues often results in severe postoperative pain, a long recovery time, and low spinal function rates [6–8].

Recently, a minimally invasive-TLIF (MI-TLIF) technique has been described, with advantages such as reduced muscular dissection resulting in smaller wounds, less tissue trauma, and faster recovery [9–11]. Interestingly, it has been demonstrated that in comparison to open-TLIF, MI-TLIF achieves the same clinical efficacy but causes less blood loss and allows an earlier return to work [9, 12, 13]. However, MI-TLIF prolongs X-ray exposure time, as an increased use of fluoroscopy is required for placement of the tubular retractor system and pedicle screws. In addition, MI-TLIF is technically challenging and requires greater familiarity with anatomy due to the limited visibility and restricted working space [14]. The long fluoroscopy time [15] and steep learning curve [16] associated with MI-TLIF limit its adoption and use by surgeons.

Therefore, there is a need to decrease the radiation exposure and technical demands of MI-TLIF procedures. Previous reports have indicated that MI-TLIF reduces intraoperative radiation exposure and fluoroscopic time during pedicle screw placement when compared with open-TLIF [17, 18]. Furthermore, it was suggested that modified MI-TLIF techniques may further improve TLIF efficacy and safety and make the procedure easier to learn [14].

Herein, we report a modified MI-TLIF procedure using a trans-multifidus approach, in which surgery was assisted by microscopy, and muscles were protected by a tunnel and retractors (MMI-TLIF). The new technique is expected to decrease the operation and fluoroscopy times, and make the technique easier for surgeons to learn, particularly for those already familiar with conventional open-TLIF. This study aimed to compare the clinical outcomes between MMI-TLIF and open-TLIF.

## Methods

### Study design

This study was approved by the ethics committee of Anhui Medical University, and patients who underwent MMI-TLIF or open-TLIF at the Anhui provincial hospital between 2008 and 2010 were included. Indications for surgery included grade 1 or 2 spondylolisthesis or degenerated discs in patients presenting with mechanical low back pain and radicular symptoms. All patients had tried conservative therapies for at least 3 months, but radicular pain was refractory to treatments. MMI-TLIF patients were matched with open-TLIF individuals based on age, sex, and spinal segments operated. Patients with previous spinal instrumentation, tumor spinal pathologies, spinal infections, or acute spinal trauma were excluded.

Patient demographic and operative data were collected. Then, signs and symptoms, neurological disorders, and radiological images were evaluated. Radiological examination included assessment of static (anterior-posterior and lateral) and dynamic (flexion and extension) plain lumbar spine radiographs, magnetic resonance imaging (MRI), and/or computed tomography (CT).

A total of 98 patients underwent MMI-TLIF (*n* = 49) or open-TLIF (*n* = 49). All patients were prospectively evaluated by independent surgeons for North American Spine Society (NASS) scores for back pain/disability and neurogenic symptoms, the Oswestry Disability Index (ODI), Visual Analogue (VAS) scores for back pain and leg pain, and Short Form-36 (SF-36) scores before surgery and during follow-up (6 months and 2 years after surgery). Fusion rates were assessed at 2 years according to a four-type scale as described by Bridwell et al. [19].

Statistical analysis was performed using SPSS 17.0 (IBM, Armonk, NY, USA). All data were tested for normality, and we then used the appropriate statistical methods. Normally distributed data are expressed as mean ± SD and were compared using the paired samples *t* test; otherwise, data were expressed as median (range) and were compared using the Wilcoxon rank sum test. The *χ*^2^ test was used to evaluate differences in categorical data. *P* < 0.05 was considered statistically significant.

### Surgical techniques

#### MMI-TLIF

The MMI-TLIF procedure was performed on the patient’s more symptomatic side, i.e., the side more severely affected was operated when both legs were symptomatic.

A midline 4-cm skin incision was made. The thoracolumbar fascia was incised longitudinally, close to the supraspinous ligament. The interfascial plane was dissected between the thoracolumbar and superficial fasciae of the multifidus muscle. The fascia of the multifidus muscle was incised 2–3 cm lateral to the midline. The multifidus and the longissimus muscles were bluntly dissected using a fingertip, and the lateral aspect of the facet joint was exposed. Muscles beside the approach were carefully protected by retractors, and vertebrae were instrumented with pedicle screws. The ipsilateral open side was decompressed, and pedicle screws were instrumented on the contralateral side. The position of the pedicle screws was verified using anteroposterior and lateral fluoroscopy. The facet joint of the ipsilateral side was exposed and a tunnel (Viper, Johnson & Johnson, USA) was set on it. A microscope (Leica, Germany) was positioned to facilitate visualization through the tunnel. The inferior articular processes of the upper vertebrae and the superior articular processes of the lower lumbar vertebrae were excised using an osteotome and a Kerrison rongeur, respectively.

The neural foramen was exposed, and the bone was harvested through the tunnel. The safety triangle zone between the exiting and traversing nerve roots was exposed, and the disc space in this area was opened. The degenerative disc was removed and the endplate prepared. Bone chips were packed in the anterior space, and an adequately sized titanium cage (Concorde Bullet, Johnson & Johnson) was placed centrally in the intervertebral space. Fluoroscopy was used to ensure satisfactory placement of the cage.

#### Open-TLIF

An incision was made through the midline skin, the fascia was incised, and the paravertebral muscles were dissected from the spine. C-arm fluoroscopy was used to determine the involved pedicles. Bilateral pedicle screw-rod constructs were inserted. The lamina was partly removed, and the facetectomy was performed. After carefully pulling the nerve root towards the midline, discectomy was performed, and a bone graft and interbody cage were inserted. Local autogenous bone was used for bone grafting. The wound was irrigated and closed carefully in layers.

#### Instrumentation and equipment

For MMI-TLIF, pedicle screw-rod instrumentations used were Monarch (59.2 %; Fule, Beijing, China) and Moss Miami (40.8 %; DePuy Spine, USA), while Concorde (DePuy Spine) interbody cages were inserted.

In the case of open-TLIF, Monarch (53.1 %; Fule) and Moss Miami (46.9 %; DePuy Spine) were used as pedicle screw-rod instrumentations, and interbody cages were Moss Miami (72.6 %; DePuy Spine) and Leopard (27.4 %; DePuy Spine).

## Results and discussion

### Patient demographic data

There were no significant differences in age, gender, body mass index, and spinal segments operated upon using MMI-TLIF or open-TLIF (*P* > 0.05). Median age in MMI-TLIF and open-TLIF patients was 49.2 (range 21.2–75.3) and 51.1 (range 22.1–72.9) years, respectively. Each group was composed of 27 females and 22 males. Median body mass indices of 24.3 (range 18.9–29.5) and 24.7 (18.3–31.7) kg/m^2^ were observed in the MMI-TLIF and open-TLIF groups, respectively. In either group, 30 patients were operated at L4–5, 12 at L5–S1, and 7 at L3–L4 (Table 1). Each group was composed of 23 patients with grade 1 or 2 spondylolisthesis and 26 patients with degenerated discs presenting with mechanical low back pain and radicular symptoms (*P* > 0.05).

**Table 1:** Patient demographic and clinical data

	MMI-TLIF (*n* = 49)	Open-TLIF (*n* = 49)
Age (years)	49.2 (21.2–75.3)	51.1 (22.1–72.9)
Gender (males)	22	22
BMI	24.3 (18.9–29.5)	24.7 (18.3–31.7)
Segment operated		
L4–L5	30	30
L5–S1	12	12
L3–L4	7	7

*BMI* body mass index, *MMI-TLIF* modified minimally invasive transforaminal lumbar interbody fusion, *Open-TLIF* open transforaminal lumbar interbody fusion

### Clinical data

The median fluoroscopy time for the MMI-TLIF and open-TLIF groups was not significantly different (15.8 vs. 12.3 s, *P* > 0.05). However, the operative time was significantly longer in MMI-TLIF patients than the open-TLIF group (91.3 vs. 82.5 min, *P* < 0.05). The median blood loss was significantly less in the MMI-TLIF group than the open-TLIF patients (75.3 vs. 215.2 ml, *P* < 0.05) (Table 2). No MMI-TLIF patient required blood transfusion, while five (10.2 %) patients in the open-TLIF group needed transfusion (median of 1.5 U, range 1–4 U). In addition, median hospitalization time was significantly shorter in the MMI-TLIF group than the open-TLIF group (3.7 vs. 6.9 days; *P* < 0.05).

**Table 2:** Perioperative parameters

	MMI-TLIF (*n* = 49)	Open-TLIF (*n* = 49)
Fluoroscopy time (sec)*	15.8 (10–42)	12.3 (8–36)
Blood loss (ml)*	75.2 (52–128)	215.2 (175–650)
Duration of surgery (min)*	91.3 (85–135)	82.5 (75–112)
Length of hospitalization (days)*	3.7 (3–7)	6.9 (5–12)
VAS: Immediate postop/on discharge*	5.0 (4–7)/1.7 (1–5)	6.0 (4–9)/2.8 (1–6)
Ambulation: room/ward (postoperative day)*	1.4 (1–2)/2.3 (2–3)	3.0 (2–4)/4.1 (3–6)
Total morphine (PCA, mg)*	15.1 (12–20)	33. (25–42)

*MMI-TLIF* modified minimally invasive transforaminal lumbar interbody fusion,

*P < 0.05

Based on VAS scores, patients in the MMI-TLIF group suffered significantly less pain compared with those in the open-TLIF group (immediate postoperation, 5.0 vs. 6.0; on discharge, 1.7 vs. 2.8; *P* < 0.05). These changes in VAS pain scores after surgery were higher than the minimal clinically important difference of 1.0–1.3 cm that has been previously reported [20, 21]. In addition, the difference of 1.1 between the two groups after surgery was borderline clinically significant. Furthermore, MMI-TLIF patients ambulated significantly faster (room, 1.4 vs. 3 days; ward, 2.3 vs. 4.1 days; *P* < 0.05) and required significantly less morphine (15.1 vs. 33.1 mg; *P* < 0.05), compared with open-TLIF patients (Table 2).

The NASS scores for back pain/disability and neurogenic symptoms, ODI scores, VAS scores, and SF-36 scores were recorded and analyzed. Interestingly, all scores were significantly improved at 6 months and 2 years postsurgery, in both groups, when compared with their respective preoperative values (Tables 3, 4, and 5). However, no significant differences between MMI-TLIF and open-TLIF groups were observed for these scores at any time point.

**Table 3:** Pre- and postoperative ODI scores for MMI-TLIF and open TLIF

ODI	MMI-TLIF (*n* = 49)	Open-TLIF (*n* = 49)
Preoperative	51 (40–60)	52 (46–59)
6 months postoperative	20 (10–32)	19 (9–30)
2 years postoperative	15 (6–21)	16 (9–23)

*ODI* Oswestry Disability Index, *MMI-TLIF* modified minimally invasive transforaminal lumbar interbody fusion, *Open-TLIF* open transforaminal lumbar interbody fusion

*P* > 0.05 when MMI-TLIF compared to open-TLIF at preoperation

*P* < 0.001 when MMI-TLIF 6 months and 2 years postoperation compared to preoperation

*P* > 0.05 when MMI-TLIF compared to open-TLIF at 6 months postoperation

*P* < 0.001 when open-TLIF 6 months and 2 years postoperation compared to preoperation

*P* > 0.05 when MMI-TLIF compared to open-TLIF at 2 years postoperation

**Table 4:** North American spine society score

	Preoperative	6 months	2 years
OPEN-TLIF BDS	3 (2–7)	2 (1–4)	1 (0–3)
MMI-TLIF BDS	3 (2–6)	2 (0–5)	1 (0–3)
OPEN-TLIF NSS	4 (3–6)	2 (1–4)	1 (0–4)
MMI-TLIF NSS	3 (2–5)	2 (0–4)	1 (0–2)

*BDS* back pain and disability score, *NSS* neurogenic symptom score, *MMI-TLIF* modified minimally invasive transforaminal lumbar interbody fusion, *Open-TLIF* open transforaminal lumbar interbody fusion

*P* < 0.01 when preoperation compared to 6 month and 2 years, respectively in both MMI-TLIF and open-TLIF groups of BDS and NSS

*P* > 0.05 when 6 months compared to 2 years in both MMI-TLIF and open-TLIF groups of BDS

*P* < 0.01 when 6 months compared to 2 years in both MMI-TLIF and open-TLIF groups of NSS

*P* > 0.05 when OPEN compared to MMI-TLIF in preoperation, 6 months and 2 years of BDS and NSS

**Table 5:** VAS back pain and leg pain score

	Preoperative	6 months	2 years
OPEN-TLIF BP	6.5 ± 2.53	1.7 ± 1.33	1.2 ± 0.71
MMI-TLIF BP	6.1 ± 2.19	1.8 ± 1.38	1.0 ± 0.93
OPEN-TLIF LP	6.5 ± 1.88	2.1 ± 1.05	1.1 ± 0.86
MMI-TLIF LP	7.2 ± 1.92	2.1 ± 1.45	1.0 ± 0.76

*BP* back pain, *LP* leg pain, *MMI-TLIF* modified minimally invasive transforaminal lumbar interbody fusion, *Open-TLIF* open transforaminal lumbar interbody fusion

*P* < 0.01 when preoperation compared to 6 months and 2 years, respectively, in both MMI-TLIF and open-TLIF groups of BP and LP

*P* > 0.05 when 6 months compared to 2 years in open-TLIF group of BP

*P* < 0.05 when 6 months compared to 2 years in MMI-TLIF group of BP

*P* < 0.01 when 6 months compared to 2 years in both open-TLIP and MMI-TLIF groups of LP

*P* > 0.05 when open-TLIF compared to MMI-TLIF in preoperation, 6 months and 2 years of BP and LP

When fusion rates were evaluated according to the Bridwell anterior fusion grading system [19], no significant differences were observed between MMI-TLIF and open-TLIF patients. Indeed, there were no grade 3 or 4 fusions in either experimental group; 81.7 and 87.8 % of MMI-TLIF and open-TLIF patients, respectively, achieved grade 1 fusion (*P* > 0.05) (Table 6).

**Table 6:** Fusion rates based on Bridwell classification

Bridwell grade of fusion	MMI-TLIF (*n* = 49)	Open-TLIF (*n* = 49)
Grade I, *n* (%)	40 (81.7)	43 (87.8)
Grade II, *n* (%)	9 (18.3)	6 (12.2)

*MMI-TLIF* modified minimally invasive transforaminal lumbar interbody fusion, *Open-TLIF* open transforaminal lumbar interbody fusion

Importantly, the complication rate for open-TLIF patients (12.1 %) was significantly higher than that in the MMI-TLIF group (12.1 vs.7.5 %; *P* < 0.05). Two MMI-TLIF patients experienced delayed wound healing, one open-TLIF patient developed urinary tract infection, and three open-TLIF diabetic patients suffered from wound infection. These patients were treated with antibiotics and all patients recovered, with no severe complications observed in either group.

We report herein a modified MI-TLIF (MMI-TLIF) technique using a trans-multifidus approach in which surgery was assisted with a microscope. The surgeon was not exposed to the radiation of C-arm fluoroscopy during the entire operation. Indeed, surgeons were shielded by a lead wall during fluoroscopy. Due to the modifications, MMI-TLIF required a median fluoroscopy time of 15 s to ensure that the correct segment was located and the pedicle screw and cage positions were accurate. The operation time of MMI-TLIF (91.3 min) was longer than open-TLIF (82.5 min), but still starkly shorter than the 210 min reported for MI-TLIF [22–24]. These findings indicate that the newly developed MMI-TLIF represents an improvement from both open-TLIF and MI-TLIF and will benefit patients as well as surgeons.

Importantly, the trans-multifidus approach reduces the technical difficulty encountered with the MI-TLIF procedure. Indeed, the trans-multifidus approach is performed through a natural cleavage plane [25], which preserves the natural posterior tension band created by the inter- and supraspinous ligaments. In addition, the muscular attachments of the paraspinous musculature on the posterior elements are also preserved. Since the pedicle screw is inserted under direct sight, the procedure is familiar to surgeons accustomed to traditional open-TLIF. Therefore, MMI-TLIF saves time and is easier to learn, which promotes its use by professionals and presents clinical advantages.

For instance, blood loss was significantly lower in the MMI-TLIF group than the open-TLIF group since the muscle was protected in MMI-TLIF patients by a retractor, which resulted in reduced blood loss from the muscle. Microscopic observation indicated that the veins around the nerve roots were well protected or coagulated, which further prevented blood loss. Therefore, blood loss in MMI-TLF patients (42 ml) was reduced, in comparison with values reported for MI-TLIF (81.5 ml) [26]. This represents an advantage over MI-TLIF and should be further explored in future studies.

The microscope constituted a powerful tool that allowed observation of details of the spinal canal structure through the tunnel. This significantly reduced the risk of neural element traction injury. The complete exposure of the far lateral aspect of the intervertebral disc space was provided by facetectomy through the retractor, and little retraction was required from the thecal sac and/or nerve roots when the interbody graft was placed. Consequently, no cases of nerve root damage were observed in patients who underwent MMI-TLIF in the present study.

The cost of treatment is an important factor that concerns many healthcare providers and patients [27, 28]. MMI-TLIF resulted in decreased number of bed days and reduced length of hospital stay, which lower costs and decrease risk of infection. Indeed, MMI-TLIF patients recovered fast and required less morphine, and inpatient rehabilitation was not needed. These properties contribute to further reduce care costs. Since the muscle of the posterior column is preserved with MMI-TLIF, patient’s work capability during the early stages of recovery may be improved, lowering the social burden of degenerative disc disease. These findings indicate the economic advantages of the MMI-TLIF procedure.

At 6 months and 2 years postoperation, both MMI-TLIF and open-TLIF patients showed significant improvements in clinical outcomes in comparison to the values obtained preoperation. When the Bridwell anterior fusion grading system was used to assess fusion rates, no significant differences were observed between MMI-TLIF and open-TLIF patients. Precisely, 81.7 and 87.8 % patients in the MMI-TLIF and open-TLIF groups, respectively, achieved grade 1 fusion, a non-statistically significant difference. These data indicate that both techniques display similar clinical efficacy. However, a non-inferiority trial should be performed to confirm these observations.

The limitation of this study is that the instrumentation used in the two groups was not consistent. A clinical trial with consistent instrumentation is therefore required to further evaluate the safety and efficacy of MMI-TLIF. In the present study, patients with grade 1 or 2 spondylolisthesis or with degenerated discs presenting with mechanical low back pain and radicular symptoms were analyzed together. However, even if there was no difference between the two groups of patients, future studies should focus on only one condition.

## Conclusions

MM-TLIF represents a safe and efficient technique. It combines the advantages of less initial postoperative pain, early rehabilitation, shorter hospitalization, and fewer complications achieved with MI-TLIF, with the long-term clinical outcomes and high fusion rates achieved with open-TLIF. In addition, MMI-TLIF minimizes the exposure of surgeons to radiation, and this technique is easy to learn. Overall, these properties make MMI-TLIF superior to MI-TLIF and open-TLIF, which should promote its use for the treatment of degenerative disc disease.
